# Measuring F-actin properties in dendritic spines

**DOI:** 10.3389/fnana.2014.00074

**Published:** 2014-08-05

**Authors:** Mikko Koskinen, Pirta Hotulainen

**Affiliations:** Neuroscience Center, University of HelsinkiHelsinki, Finland

**Keywords:** actin, FRAP, PAGFP, FRET, dendritic spine

## Abstract

During the last decade, numerous studies have demonstrated that the actin cytoskeleton plays a pivotal role in the control of dendritic spine shape. Synaptic stimulation rapidly changes the actin dynamics and many actin regulators have been shown to play roles in neuron functionality. Accordingly, defects in the regulation of the actin cytoskeleton in neurons have been implicated in memory disorders. Due to the small size of spines, it is difficult to detect changes in the actin structures in dendritic spines by conventional light microscopy imaging. Instead, to know how tightly actin filaments are bundled together, and how fast the filaments turnover, we need to use advanced microscopy techniques, such as fluorescence recovery after photobleaching (FRAP), photoactivatable green fluorescent protein (PAGFP) fluorescence decay and fluorescence anisotropy. Fluorescence anisotropy, which measures the Förster resonance energy transfer (FRET) between two GFP fluorophores, has been proposed as a method to measure the level of actin polymerization. Here, we propose a novel idea that fluorescence anisotropy could be more suitable to study the level of actin filament bundling instead of actin polymerization. We validate the method in U2OS cell line where the actin structures can be clearly distinguished and apply to analyze how actin filament organization in dendritic spines changes during neuronal maturation. In addition to fluorescence anisotropy validation, we take a critical look at the properties and limitations of FRAP and PAGFP fluorescence decay methods and offer our proposals for the analysis methods for these approaches. These three methods complement each other, each providing additional information about actin dynamics and organization in dendritic spines.

## Introduction

Dendritic spines are small bulbous protrusions from the dendritic shafts of neurons. These distinct cellular compartments house the majority of the postsynaptic terminals of excitatory synapses. Synaptic function has a strong coupling to the morphology and dynamics of dendritic spines (Alvarez and Sabatini, 2007; Bhatt et al., 2009). The plasticity of the synaptic terminals found in dendritic spines enables them to shape the function of the neuronal network. This has led to the hypothesis that dendritic spines are the sites of memory formation and maintenance in the brain (Kasai et al., 2010; Chen et al., 2014). Abnormalities in the shape and size or the development and distribution of dendritic spines have been linked to several neurological diseases such as Autism spectrum disorders (ASD), Schizophrenia, Alzheimers disease (AD) (Penzes et al., 2011), Down syndrome and mental retardation (Kaufmann and Moser, 2000).

The main cytoskeletal component of dendritic spines is actin (Hotulainen and Hoogenraad, 2010). Dendritic spines are enriched with actin, containing approximately 6 times more actin than dendritic shafts (Honkura et al., 2008). Actin monomers (globular actin, G-actin) polymerize to form actin filaments (filamentous actin, F-actin). Most of the actin in dendritic spines is F-actin with only about 12% of the total actin being monomeric (Star et al., 2002; Honkura et al., 2008). Single actin filaments undergo constant exchange of monomers from the ends of the filaments. The filaments are polar containing a barbed (plus)-end and a pointed (minus)-end. Polymerization and depolymerization rates for the ends are different leading to a constant flow of actin monomers through the filaments from barbed to pointed ends. This process is called treadmilling (Pollard and Cooper, 2009). Actin filaments can control cell shape by exerting a mechanical force on the cell membrane through polymerization (Pollard and Cooper, 2009; Blanchoin et al., 2014).

F-actin structures range from branched filament network to thick actin bundles of several cross-linked filaments (Blanchoin et al., 2014). These structures have highly variable lifetimes. Filaments forming the actin mesh at the leading edge of a migrating cell can change very rapidly whereas thick actin bundles called stress fibers can remain stable for a long time. The three-dimensional structure and dynamics of actin filament network are regulated by actin binding proteins (ABPs) (Dos Remedios et al., 2003; Pollard and Cooper, 2009). ABPs can affect the treadmilling rate of the filaments as well as their stability and organization. ABPs have a multitude of different functions; they can initiate polymerization, induce branching of filaments, cap filaments and block polymerization, sever filaments to shorter pieces, depolymerize filaments, bundle or cross-link filaments, produce contractile force or protect filaments from depolymerization (Dos Remedios et al., 2003).

A number of studies have investigated the roles of different ABPs in regulating the dynamics and organization of dendritic spine actin (Hotulainen and Hoogenraad, 2010). Numerous ABPs have been shown to influence neurophysiological function (Lamprecht, 2014). For example, myosin IIb, which binds actin and can induce contractility into a network, has been indicated in long-term potentiation (LTP) stabilization and memory consolidation (Rex et al., 2010). The loss of Arp2/3, the protein complex known for nucleating actin filaments and the induction of branched actin filament network, has been shown to lead to cognitive, psychomotor and social disturbances in aging mice (Kim et al., 2013). Cofilin-1, which severs and depolymerizes actin filaments, has been suggested to be involved in late-LTP (L-LTP) induction and associative learning (Rust et al., 2010) as well as in memory extinction (Wang et al., 2013). In line with these results, defects in the regulation of the actin cytoskeleton in neurons have been implicated in memory disorders (Penzes et al., 2011; Caroni et al., 2012).

In order to be able to study the dendritic spine F-actin organization and dynamics we need to have suitable microscopy techniques. Different actin structures can be relatively easily visualized by conventional fluorescent microscopy techniques in fibroblasts. However, the average size of dendritic spines themselves is close to the physical resolution limit of conventional light microscopy and therefore more advanced light microscopy techniques or electron microscopy methods are necessary to image and measure actin organization and dynamics in these compartments. With electron microscopy, actin spinoskeleton has been shown to consist of a combination of short and branched filaments and longer bundles of actin filaments (Korobova and Svitkina, 2010). However, visualizing actin filaments by electron microscopy approaches is feasible only in a few laboratories. In addition, electron microscopy is possible only with fixed samples and is thus not applicable to measure actin dynamics. Therefore, several light-microscopy methods have been applied to measure the properties of actin filaments in dendritic spines. These techniques involve fluorescence recovery after photobleaching (FRAP), photo activated green fluorescent protein (PAGFP) fluorescence decay, photo-activation localization microscopy (PALM), Förster resonance energy transfer (FRET) and stimulated emission depletion (STED) fluorescence microscopy.

FRAP and PAGFP decay experiments have shown that spine F-actin can be divided into two pools based on their turnover rate: the fast turnover rate dynamic pool with a time constant <1 min and a slower turnover rate stable pool with a time constant of ~17 min (Star et al., 2002; Honkura et al., 2008). Honkura and colleagues also detected an enlargement pool that formed into dendritic spines following LTP induction. LTP stabilization was found to be dependent on the retention of the enlargement pool of actin inside the dendritic spine head (Honkura et al., 2008). Single molecule tracking (PALM) has revealed that actin filaments in dendritic spines are relatively short (~200 nm) and that they exhibit very heterogeneous treadmilling rates (Frost et al., 2010).

The organization of actin filament structures also plays an important role in the regulation of actin filament turnover rate as well as in determining the overall function of the spinoskeleton. Conventional two-color FRET experiments have revealed that LTP formation causes a shift of F-actin/G-actin equilibrium toward F-actin (Okamoto et al., 2004). Recently, a fluorescence anisotropy-based FRET method (only one fluorophore i.e., homoFRET) was proposed as an alternative way to probe actin polymerization state (Vishwasrao et al., 2012). It is important to note that fluorescent molecules are typically expressed in very low levels in neurons. The probability for FRET to occur drops to less than 1% when the fluorophore pair is further than 10 nm apart. If every tenth actin monomer in a single filament is coupled to GFP, there would be GFP in every fifth monomer in an actin double helix. This distance is already ~20 nm, meaning that GFP molecules are too far away from each other to carry out FRET. For this reason, we wanted to test whether the fluorescence anisotropy reflects the inter-filament rather than intra-filament FRET. In other words, if fluorescence anisotropy can be used to measure the level of F-actin bundling instead of the level of F-actin polymerization.

In addition to fluorescence anisotropy validation, we take a critical look at the properties and limitations of FRAP and PAGFP fluorescence decay methods and offer our proposals for the optimal application of these approaches. These three methods complement each other, each providing additional information about actin dynamics and organization. Optimally these methods could be used together or in combination with super-resolution approaches to improve our understanding on the organization and dynamics of the dendritic spine actin filaments.

## Materials and methods

### Neuronal cell cultures, transfection and fixed sample preparation

Rat hippocampal neuron cultures were prepared as described previously (Bertling et al., 2012). Briefly, the hippocampi of embryonal day 17 Wistar rat fetuses were dissected and the meninges were removed. The cells were dissociated with 0.05% papain along with mechanical trituration. The plating density of the cells was 1,00,000 cells/coverslip (diameter 13 mm). The coverslips were coated with Poly-L-Lysine (0.1 mg/ml) (Sigma). Cells were plated in neurobasal medium (Gibco) supplemented with B-27 (Invitrogen), L-glutamine (Invitrogen) and penicillin-streptomycin (Lonza). Transfections were performed as described previously (Hotulainen et al., 2009) at days *in vitro* (DIV) 10–13 or DIV20 using Lipofectamine 2000 (Invitrogen). Plasmids pEGFP-N1 (GFP) and mCherry-C1 (mCherry) were purchased from Clontech Laboratories, Inc. Human GFP-β-actin (GFP-actin) (Choidas et al., 1998), PAGFP-actin and GFP-GFP (Dopie et al., 2012) were gifts from Maria Vartiainen (University of Helsinki, Finland). mCherry-palladin was a gift from Gergana Gateva and Pekka Lappalainen (University of Helsinki, Finland). mCherry-MHCIIb and mCherry-MHCIIb-R709C were kind gifts from Alan Rick Horwitz (University of Virginia, USA). siRNA oligonucleotides against MHCIIb (target sequence CCGGGATGAAGTGATCAAGCA) were purchased from Ambion. U2OS cells were cultured, plated and transfected as described earlier in Hotulainen and Lappalainen (2006). Cells were fixed with 4% PFA. F-actin was stained using phalloidin conjugated to Alexa 594 (dilution 1:400, Molecular Probes).

### Confocal imaging

Confocal imaging was performed with Leica TCS SP5 or Leica SP5 MP SMD FLIM upright confocal microscopes. For fixed sample imaging a 63 × 1.3 NA objective lens was used. Live-cell recordings were performed using a 63 × 0.9 NA water-dipping objective. For all live-cell experiments the microscopes were equipped with temperature controlled chamber and CO_2_ supply. Live-cell experiments were performed at 37°C and 5% CO_2_. The image files were processed with LAS AF (Leica microsystems, Germany), Photoshop CS6 (Adobe) and ImageJ softwares.

### FRAP

FRAP experiments were performed as described previously (Koskinen et al., 2012). Briefly, neurons were transfected with GFP-actin at DIV13. Only mature mushroom type spines were used for the experiments. The frame including the ROI (measured whole spine) was imaged three times before bleaching. Photo-bleaching was achieved with five scans (total bleach time 3.117 s) of the region of interest with ~2.2 mW laser power at the sample (488 nm). Imaging of the area was resumed immediately after photo-bleaching and continued every 2–20 s for ~100–300 s. All the post-bleach values were divided by the values from the non-bleached area of the cells and normalized to the first pre-bleach value. The first post-bleach measurement was set to 0 s. The analysis of the FRAP recovery data was performed as described in the text using LAS AF (Leica Microsystems, Germany), Excel (Microsoft) and Origin8.6 (OriginLab) software.

### PAGFP fluorescence decay

PAGFP fluorescence decay experiments were performed as described previously (Koskinen et al., 2012). Briefly, neurons were co-transfected with PAGFP-actin and mCherry at DIV10. Only mushroom spines of similar size were used for the experiments. Photo-activation was induced with one scan (0.78 s) of moderate intensity (~0.4 mW at the sample) 405 nm laser over the ROI (a single spine). Imaging of the area was resumed immediately following photo-activation and continued every 5–60 s for ~1000 s. For the analysis purposes the last pre-activation frame intensity was first subtracted from all post-activation values to exclude background and possible channel cross-talk. All post activation values were normalized to the first measured value. The activation frame was set as 0 s. For illustration figures the contour images were acquired from mCherry images by using Matlab 2010a (Mathworks, US). The data were processed with a 3 × 3 medial filter to reduce the noise. After this, the images were intensity-thresholded at a value of 25 to remove the background. The morphological “open” operation was performed on the resulting binary image to further smooth the edges. Finally the edges were calculated from these binary images using Canny edge detector (Canny, 1986). The analysis of the data was performed with LAS AF (Leica Microsystems, Germany), Excel (Microsoft) and Origin8.6 (OriginLab) Software as described in the text and figure and table legends.

### Fluorescence anisotropy

Förster resonance energy transfer between two GFP-molecules, i.e., homoFRET, was quantified by measuring the fluorescence anisotropy. The experiments were carried out with a Leica SP5 MP SMD FLIM microscope (Leica Microsystems, Germany) using a 63 × 0.9 NA dipping objective. An argon laser (488 nm) was used to excite the fluorophores. A polarization prism (Leica Microsystems, Germany) was used to divide the emission signal into two channels, one parallel and the other perpendicular to the excitation laser. Both signals were measured using MPD PDM/C single-photon avalanche diodes (PicoQuant GmbH, Germany) and a photon counting mode to accurately measure the intensity variations within the sample.

The data were processed with Las AF software (Leica Microsystems, Germany) and Excel (Microsoft) using the equation presented by Bader et al. (2011):

$$r_{ani}=\frac{I_{∥}-I_{⊥}}{I_{∥}+2I_{⊥}}$$

Equation 1: Anisotropy at a given pixel, or any given region of interest, is calculated as a ratio of detected emission on channels measuring light parallel and perpendicular to the excitation laser.

Average anisotropies were calculated from either whole cell images of U2OS cells transfected with GFP, GFP-GFP, GFP-Actin, or GFP-actin + palladin-mCherry or from selected regions of interest (ROIs) from the images of spines acquired from neurons transfected with free GFP, GFP-actin (at DIV14 and DIV21) or GFP-actin together with myosin heavy chain IIb WT-mCherry (MHC WT), MHC IIb siRNA or a non-contractile mutant MHC IIb R709C-mCherry (MHC IIb R709C). Anisotropy images shown in Figures 6C,E were calculated from the raw data as in equation 1, and subsequently processed with a 3 × 3 median filter to reduce noise. The resulting images were thresholded so that any values smaller than 0.1× the brightest pixel were set to zero. This processing was carried out with Matlab software (MathWorks, USA).

### Statistical tests

Statistical analyses were performed with SPSS software package (IBM). A paired sample *t*-test, Mann–Whitney test and ANOVA were used as applicable. All values in the text and figures represent mean ± standard deviation (SD) unless otherwise indicated.

## Measuring actin turnover rate in dendritic spines

The active turnover of actin structures is a fundamental property of the filaments. Many of the ABPs function by manipulating the actin turnover rate to regulate different cellular processes such as cell division, cell motility (Pollard and Cooper, 2009), spinogenesis (Hotulainen et al., 2009) and spine morphology (Hodges et al., 2011). Therefore, measuring the actin turnover rate provides an important insight into the roles of different ABPs in dendritic spines. In the following paragraphs, we describe how actin turnover rate can be measured with FRAP and PAGFP fluorescence decay. We discuss what parameters should be taken into account when these methods are applied, possible obstacles to the application of these methods and finally the analysis of the data.

### Fluorescence recovery after photobleaching (FRAP)

FRAP is the most commonly used bulk kinetics approach method to study the dynamics of actin structures. In FRAP, a fluorescent protein is bleached from a small volume inside the cell with high-power laser. The recovery of the fluorescence is followed by time-lapse imaging. The fluorescence recovery is then used to elucidate the dynamics of the protein of interest (Star et al., 2002; Hotulainen et al., 2009; Koskinen et al., 2012; Kim et al., 2013; Stamatakou et al., 2013). When using FRAP to measure actin turnover, the recovery of the fluorescence represents addition of new non-bleached monomers onto the filaments and the depolymerization and diffusion of the bleached monomers. In other words, FRAP estimates the turnover rate by measuring the rate of actin filament assembly (Koskinen et al., 2012).

When using FRAP to determine the actin turnover rate in dendritic spines certain assumptions are usually made which are valid when the whole spine is bleached: first, the actin filaments do not diffuse through the spine neck, and second, actin monomer diffusion in and out of the spine is fast with a time constant of under 1 s (range 5–670 ms) (Star et al., 2002; Honkura et al., 2008). Therefore, the G-actin diffusion is not a rate limiting step. As an exception to these assumptions, filament diffusion out of the spine has been observed in special cases such as in failure to stabilize LTP (Honkura et al., 2008). When the whole spine area is bleached and measured, the actin network flow (Honkura et al., 2008; Frost et al., 2010) inside the spine does not affect the results.

#### Parameters to consider when using FRAP

Key points to consider when using FRAP are a strict timescale of bleaching and recovery follow-up as well as the power of the laser used for bleaching. In general it is good to keep all settings the same throughout the experimental setup. All live-cell fluorescence imaging leads to phototoxic effects (Magidson and Khodjakov, 2013). In photobleaching, some phototoxic effects are by definition unavoidable. It is better to use a short powerful laser pulse, than to increase the bleaching time. The turnover time constant for G-actin inside the dendritic spine is <1 s. To minimize the effect on the whole cell fluorescence level, bleach times should be kept as short as possible. How long bleaching is necessary for proper bleaching depends on the imaging setup and the sample. Bleaching laser power should not be increased to levels where noticeable, acute, effects on the cell are apparent, such as no recovery of fluorescence or cell death. The shortest possible bleach time minimizes the amount of G-actin diffusing in and out of the spine during bleaching thus minimizing the effect on the initial recovery in the form of bleached monomers diffusing back into the spine. The experiments described in the following paragraphs have been carried out with 2.2 mW laser power at the sample.

#### A critical look at the properties and limitations of FRAP

**Figure 1:**
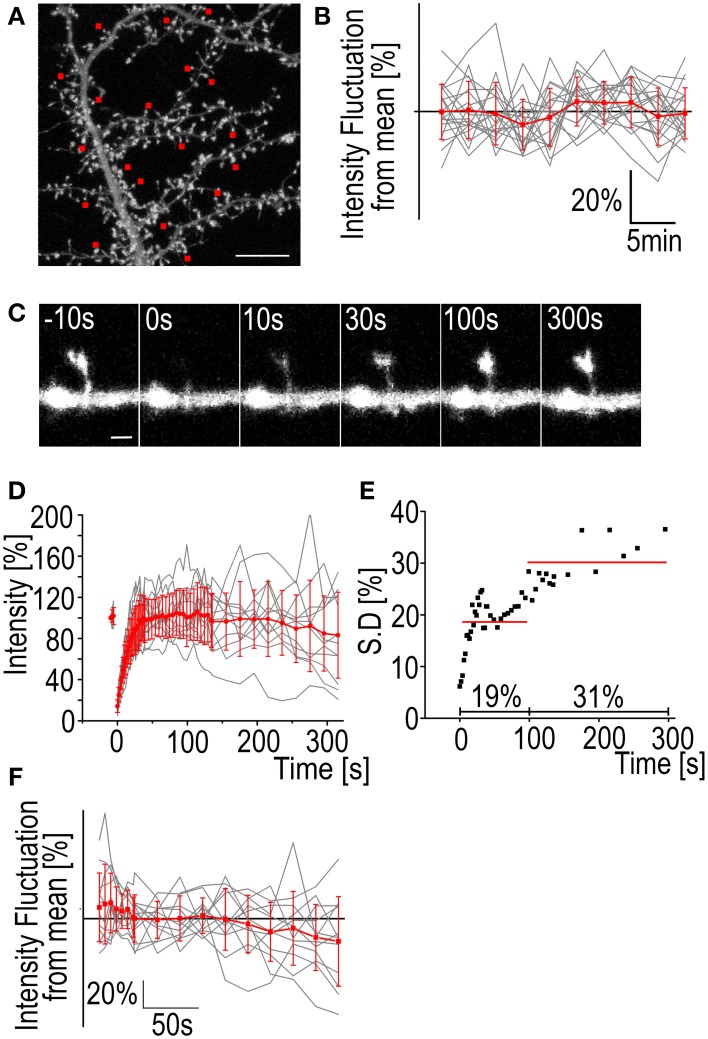
**Fluctuation of F-actin fluorescence and FRAP standard deviation**. **(A)** A maximum projection of a DIV14 rat hippocampal neuron transfected with GFP-actin. Image is the first in a 3D time-lapse series of 10 images with 3 min intervals. The red squares mark the spines whose fluorescence intensity was followed during the series. Scale bar, 10 μm. **(B)** The fluorescence intensity of spines from the hippocampal neuron represented in **(A)**. Gray traces represent the individual spines and the red trace is mean ± SD Mean spine intensity over all timepoints was set to 100%. *n* = 19 spines. **(C)** A time-lapse series of a DIV14 rat hippocampal neuron transfected with GFP-actin. The GFP fluorescence was bleached and the recovery followed. Numbers on top of the images are time after bleaching. Time *t* = 0 s is the first measurement post bleaching. Scale bar, 1 μm. **(D)** GFP-actin FRAP recovery curves from DIV14 rat hippocampal neuron spines. The gray traces represent individual spines and the red is mean ± SD, *n* = 12 spines. **(E)** The standard deviation of the mean FRAP recovery curve from **(D)**. The red lines represent the average of 19% during the first 100 s of recovery and 31% during the recovery between 100 and 315 s. **(F)** The fluorescence intensity fluctuation from the FRAP data in **(D)** after 100 s recovery. Mean spine intensity over all timepoints was set to 100%. Gray traces are individual spines and the red trace is mean ± SD.

In FRAP we are measuring a change in fluorescence intensity as it recovers from the photo-bleaching. This means that any intrinsic fluctuation of the fluorescence intensity in the system is compromising the accuracy of the measurements. Dendritic spines, especially in neuron cultures, are highly dynamic (Korkotian and Segal, 2001; Bertling et al., 2012). Thus, actin filaments in the dendritic spines are continuously re-organized. This is detected as constant increase and decrease of the GFP-actin fluorescence found in nearly all spines (Figures 1A,B). On average, the GFP-actin fluorescence intensity of a single spine of a cultured rat DIV14 hippocampal neuron fluctuates at an amplitude of 8 ± 1.7% of the mean value (Figure 1B).

Bleaching of the GFP-molecules requires a considerable dose of radiation concentrated to a single spine. The effects of this radiation on the spine morphology and actin dynamics are difficult to quantify but it seems evident that bleaching increases fluorescence intensity fluctuation (and spine dynamics). If we look at the standard deviation of the actin fluorescence after bleaching we can see that the average SD of the measurements is 19% during the first 100 s of the recovery. The fluctuation becomes even greater when the recovery reaches a plateau with the average SD 100–315 s after bleaching being 31% (Figures 1C–E). The fluorescence values for single spines 100 s after bleaching fluctuate at an amplitude of 17 ± 8.1% from the mean (Figure 1F). Comparison to fluctuation of non-bleached neurons reveals that the GFP-actin intensity exhibits larger fluctuation in bleached spines (Figures 1B vs. F). Fluorescence fluctuation makes accurate determination of intensity value difficult, both before bleaching as well as after full recovery. SD especially increases after the 100 s mark. We therefore decided to image recovery for only up to 100 s after bleaching. Consequently, FRAP is not suitable to measure the properties of the stable actin pool in dendritic spines. It can be used to estimate the stable pool size but it is better to use PAGFP fluorescence decay to confirm results, especially if the change in the stable pool size between different treatments is small (see next section).

**Figure 2:**
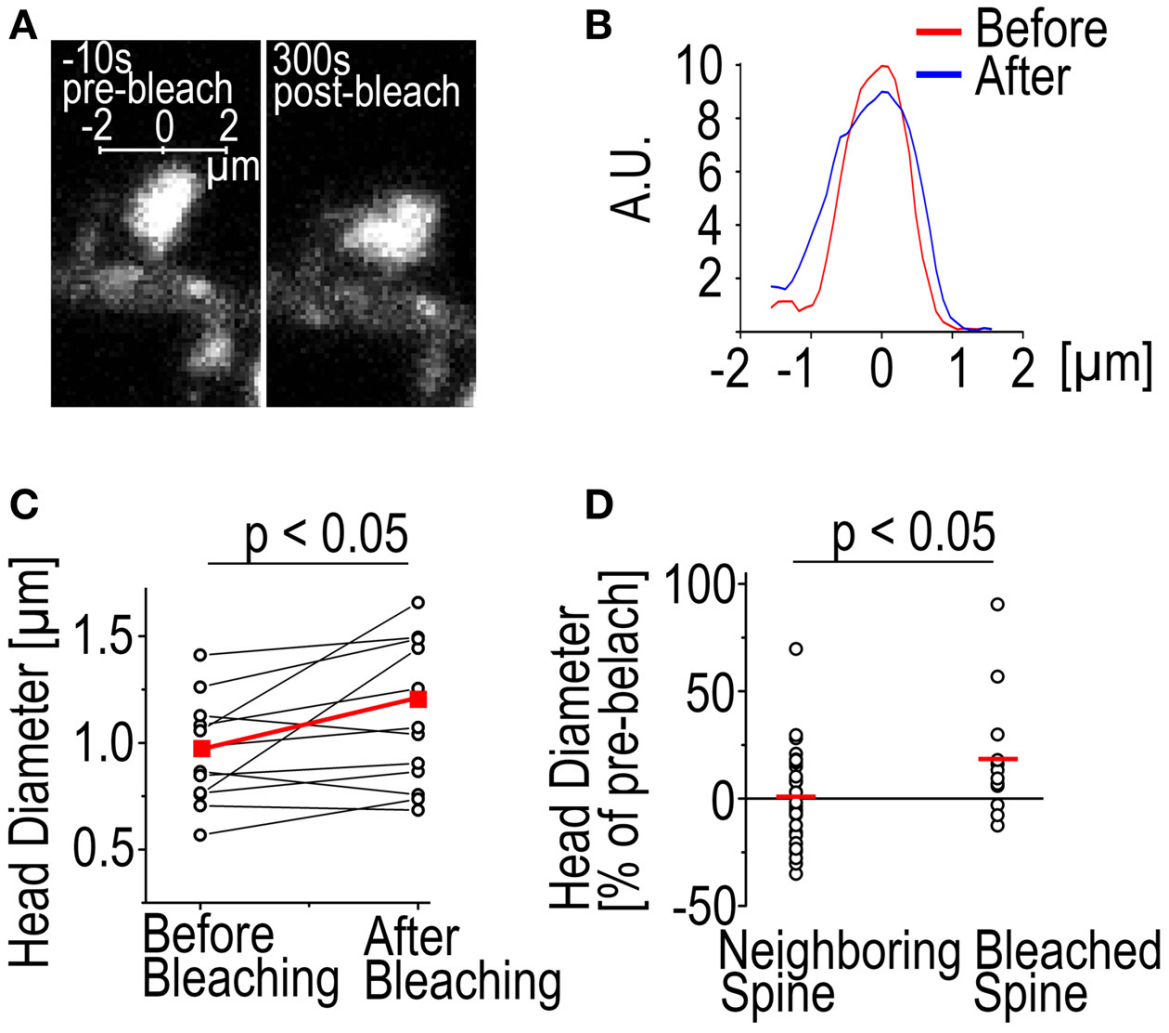
**Spine enlargement following photo-bleaching**. **(A)** A DIV14 rat hippocampal neuron spine imaged 10 s before and 300 s after photo-bleaching. **(B)** The fluorescence profiles of the spine in **(A)** through the middle of the spines along the axis shown in **(A)**. The red trace represents the profile before bleaching and the blue after bleaching. Profile was averaged over 0.5 μm along the axis. **(C)** Spine head diameter before and after bleaching. Black traces are individual spines, red trace is the mean. The mean spine size as full width at half maximum through the center of the spine changes from 0.95 ± 0.25 to 1.12 ± 0.34 μm, *p* < 0.05 Paired samples *t*-test, *n* = 12 spines. **(D)** Spine enlargement affects only the bleached spines and not the neighboring ones. Black circles represent individual spines, mean indicated by a red line. Size change: Neighboring −0.70 ± 3.6%; bleached 19 ± 29%, *p* < 0.05 Mann–Whitney test, *n* = 35 neighboring spines, 12 bleached.

Another phenomenon we have observed is the enlargement of spines following bleaching (Figure 2). In 9/12 cases the spine head was enlarged 315 s after bleaching as measured by full-width-at-half-maximum of the GFP-actin fluorescence (Figures 2A,B). The average enlargement was 27% of pre-bleach value, almost twice the average fluctuation of spine head width previously reported (Bertling et al., 2012). In 3/12 cases the spines shrunk by an average of 7.8%. Mean spine width was increased from 0.95 ± 0.25 μm to 1.1 ± 0.34 μm (*p* < 0.05 Paired samples *t*-test) (Figure 2C). In the neighboring control spines 17/35 were found enlarged. The average enlargement was 16%. The average spine shrinkage found in 18/35 cases was 17%. Counting all the spines showed that neighboring spines stayed on average the same size as before bleaching, with an average size change of −0.70 ± 3.6%, as opposed to bleached spines which were enlarged by 19 ± 29% (*p* < 0.05 Mann–Whitney test) (Figure 2D). The cause of this spine enlargement is not known but it seems that it is most obvious when a high laser power is used (our unpublished observations). So although high laser power enables the use of short bleaching time, it might have stronger undesirable effects on the cell than long bleaching with a low laser power. Spine enlargement after bleaching makes it difficult to estimate the ratio of the dynamic and stable actin pools; the amount of total actin in spines increases resulting in the fluorescence intensity overshooting the initial pre-bleach value.

Due to different laser power and different bleaching times used in different microscopy setups, it is difficult to directly compare FRAP values from different laboratories. In addition, values from different model systems (cultured neurons/ slice cultures) cannot be directly compared.

#### Analysis of FRAP data

FRAP data is usually analyzed by measuring the fluorescence at the ROI (spine) at time intervals, dividing this with a reference fluorescence value from elsewhere in the cell to correct for general fluctuation (usually bleaching caused by imaging + detector error) of the fluorescence, and normalizing to fluorescence values at the ROI before bleaching (Star et al., 2002; Koskinen et al., 2012). Several different strategies have been employed to interpret the resulting fluorescence recovery data. Most common strategies have been (a) fitting the individual measurement data to a single exponent equation (Star et al., 2002) and (b) determining the recovery half-time from averaged data and using that to calculate the first order rate constant

$$\left(k_{obs}=\;\frac{\ln(2)}{t_{1/2}}\right)$$

Equation 2. Calculation of the first order rate constant of the fluorescence recovery.

**Figure 3:**
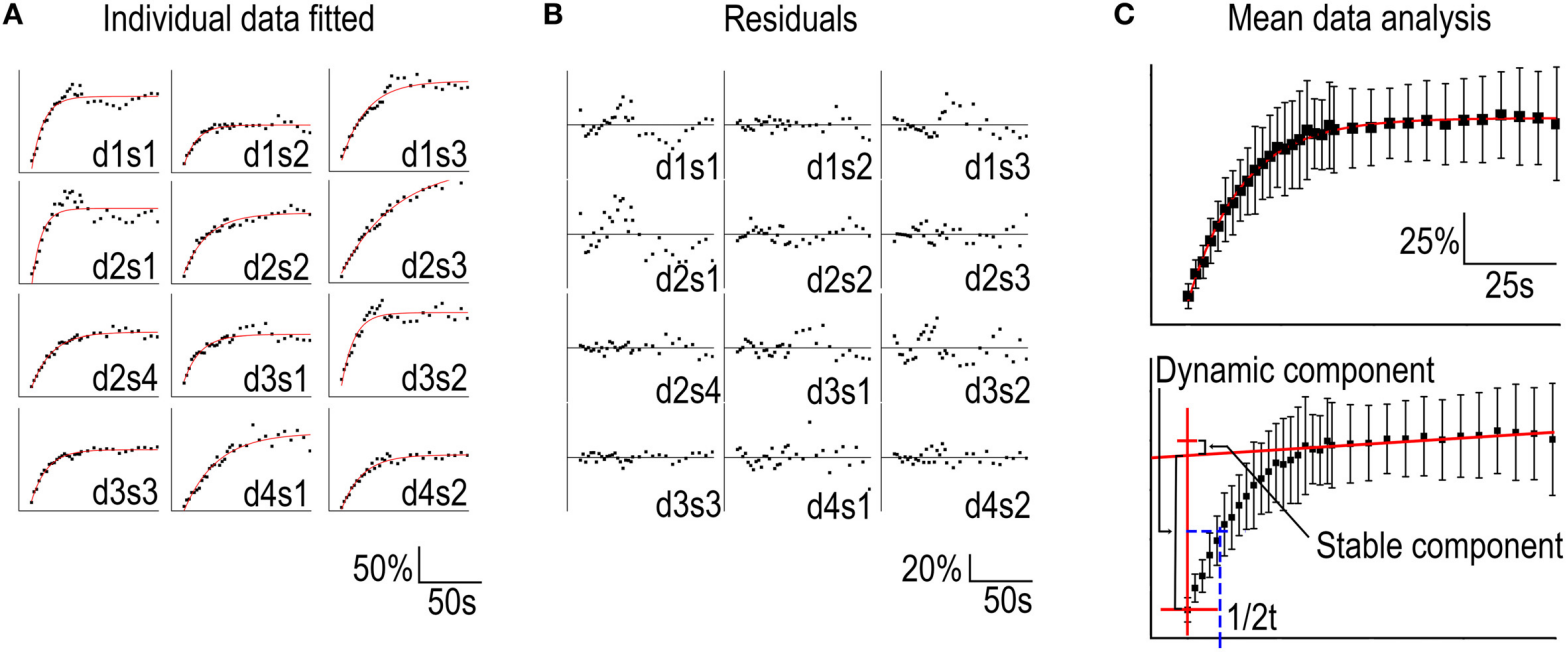
**Analysis of FRAP curves**. **(A)** Individual FRAP data measured from GFP-actin transfected, DIV14, rat hippocampal neuron spines fitted to the equation in Table 1. Data were labeled as spines (s) 1–4 belonging to a specific dendrite (d) 1–4. **(B)** The residuals corresponding to the fits in **(A)**. **(C)** Upper panel: Mean ± SD curve from data in **(A)** fitted to a single component equation as in **(A)**. Lower panel: The same mean ± SD curve as in the upper panel with the time = 0 and the linear approximation of the stable component marked by red lines and the dynamic component recovery half time in blue.

Hotulainen et al. (2009); Kim et al. (2013); Stamatakou et al. (2013); Koskinen et al. (2014). To evaluate the fitting method we used the FRAP data presented in Figure 1. We fitted each individual spine data to a single component equation (Table 1, Figure 3A).

**Table 1:** **Results of fitting curves from individual FRAP experiments to the equation $y(t)=y_{0}-A_{1}e^{-\frac{t}{t_{1}}}$ where y_0_ is the stable component, A_1_ is the dynamic component and t_1_ is the time constant, inverse of the rate constant**.

**Spine**	**y_0_ Value**	**y_0_ SE**	**A_1_ Value**	**A_1_ SE**	**t_1_ Value**	**t_1_ SE**	**Statistics Reduced Chi-Sqr**	**Statistics Adj. R-Square**
d1s1	−0.12	0.023	1.1	0.071	9.1	1.2	0.0085	0.89
d1s2	0.30	0.013	0.58	0.036	10	1.2	0.0024	0.91
d1s3	−0.32	0.034	1.2	0.054	20	2.2	0.0075	0.94
d2s1	−0.10	0.035	1.1	0.12	7.5	1.6	0.023	0.76
d2s2	−0.034	0.021	0.88	0.035	19	1.7	0.0030	0.95
d2s3	−0.73	0.060	1.6	0.052	43	3.9	0.0033	0.98
d2s4	0.059	0.013	0.81	0.027	16	1.1	0.0016	0.97
d3s1	0.091	0.020	0.68	0.047	13	1.8	0.0046	0.88
d3d2	−0.23	0.026	1.1	0.076	9.8	1.4	0.010	0.88
d3s3	0.10	0.0088	0.75	0.022	12	0.72	9.3E-04	0.98
d4s1	−0.14	0.044	1.1	0.053	26	3.3	0.0074	0.93
d4s2	0.18	0.015	0.80	0.029	16	1.3	0.0019	0.96
Average	−0.078	0.026	0.97	0.052	17	1.8	0.0061	0.92

Two features stand out from these results: (1) the wide range of recovery time constants (t_1_) and, (2) the negative stable component values (y_0_). The heterogeneity of the time constants undermines the sensitivity of the method. Our experiments resulted in a mean time constant of 17 ± 10 s with a 95% confidence interval of 11–23 s. This means that small differences in time constant are not reliably detected unless *n* is considerably large. If all conditions would follow the same distribution of time constants, these twelve measurements would be enough to acquire a statistical power for 50% change (8.5 s difference) in turnover rate. To detect a 2 s difference in mean time constants with 90% statistical power, *n* would need to exceed ~200 measurements. Thus, FRAP is only suitable for analyzing large changes in actin dynamics.

The negative values for the stable component are the result of the fluorescence intensity overshooting the initial pre-bleach values, due to intrinsic and/or bleaching-induced fluctuations in intensity and/or spine enlargement.

The signal quality of the measurements was determined by looking at the residuals from the fits (Figure 3B). Residual is the difference between the each data point and fit at that point. The smaller the range of residuals is, the better is the signal quality and fit to the used equation. The residuals of individual FRAP experiments spread roughly between −40 and 40%. The mean of all absolute residual values was 5.5 ± 5.0%.

Averaging the data from all spines before fitting leads to a better fit to single component equation (Table 2, Figure 3C) but still results in a negative stable component value.

**Table 2:** **Result of fitting average FRAP curve to the equation $y(t)=y_{0}-A_{1}e^{-\frac{t}{t_{1}}}$ where y_0_ is the stable component, A_1_ is the dynamic component and t_1_ is the time constant, inverse of the rate constant**.

	**y_0_ Value**	**y_0_ SE**	**A_1_ Value**	**A_1_ SE**	**t_1_ Value**	**t_1_ SE**	**Statistics Reduced Chi-Sqr**	**Statistics Adj. R-Square**
Average	−0.034	0.0053	0.92	0.011	15	0.38	2.82E-04	0.9956

The difficulties in determining the stable component size and distinguishing the recovery of the stable and dynamic components through automated curve fitting can be overcome by employing a simpler analysis of the mean curve. Using the mean recovery curve, the size of the stable component can be manually approximated by determining the slope of the curve segment after ~5 times the time constant of the dynamic pool. At this point > 99% of the dynamic pool fluorescence has been recovered (Figure 1D). Interpolating a straight line with this slope to the timepoint corresponding to the first post-bleach measurement gives an approximation of the stable pool size (Honkura et al., 2008) (Figure 3C, lower panel). The recovery half-time of the dynamic component can then be determined between the first measurement and the stable pool fraction and used to calculate the first order rate constant of the recovery (Figure 3C). This manual analysis of the measured values results in a stable component of 8.5% and a recovery half-time of 9.5 s which corresponds to a time constant of 14 s.

It is important to note that the stable component refers here to the actual coefficient of the immobile or slower F-actin pool. The stable pool can also be defined as a fraction of total F-actin (Star et al., 2002; Honkura et al., 2008).

$$\left(\frac{F_{stable}}{F_{stable}+F_{dynamic}}\right).$$

The main obstacle in the analyses of FRAP data was the enlargement of spines after bleaching. Obviously, spine enlargement should be minimized by optimizing bleach settings. Alternatively, the reference color (such as mCherry-actin) could be used to make it possible to correct for intrinsic fluctuation or increased actin fluorescent intensity during imaging. Using two colors is technically more challenging but can give more accurate results. Averaging whenever possible can also improve the results.

### Photo-activatable GFP fluorescence decay

Photo-activatable GFP is essentially a GFP-molecule with a point mutation resulting in a very low GFP fluorescence intensity prior to activation. Brief illumination by a 405 nm laser increases fluorescence 100 fold (Patterson and Lippincott-Schwartz, 2002). Approximation of the F-actin turnover rate using PAGFP is similar to FRAP except that the decay of the activated PAGFP fluorescence from the spine is followed (Honkura et al., 2008). At an ideal steady state, where actin polymerization and depolymerization rates are the same, FRAP and PAGFP fluorescence decay mirror one another resulting in identical time constants. However, the two methods do give different results in the presence of fluctuations in polymerization and depolymerization. For example, an increase in polymerization within the spine would be more evident in FRAP rather than in PAGFP fluorescence decay, and increase in depolymerization and filament disassembly would be more visible in PAGFP fluorescent decay. In dendritic spines, polymerization and depolymerization rates fluctuate constantly, which can also be seen in the intrinsic fluctuation of the GFP-actin fluorescence (Figures 1A,B).

#### Parameters to consider when using PAGFP fluorescence decay

When performing PAGFP-decay experiments the key points to control are: (a) the time scale, (b) the imaging settings, and (c) spine size. The stable pool size is often calculated as a fraction of the total fluorescence at the first imaged frame after PAGFP activation (Honkura et al., 2008; Koskinen et al., 2014). Therefore, the time interval from activation to the first frame has to be kept constant in order to compare different measurements. Imaging settings need to be optimized to keep the activated fluorescence in the linear range of the detector and avoid detector saturation. If planning to pool results from single spines to study the size of the stable pool, spine size must be taken into account as the size of the stable pool is correlated to spine size (Honkura et al., 2008).

#### Differences to FRAP

**Figure 4:**
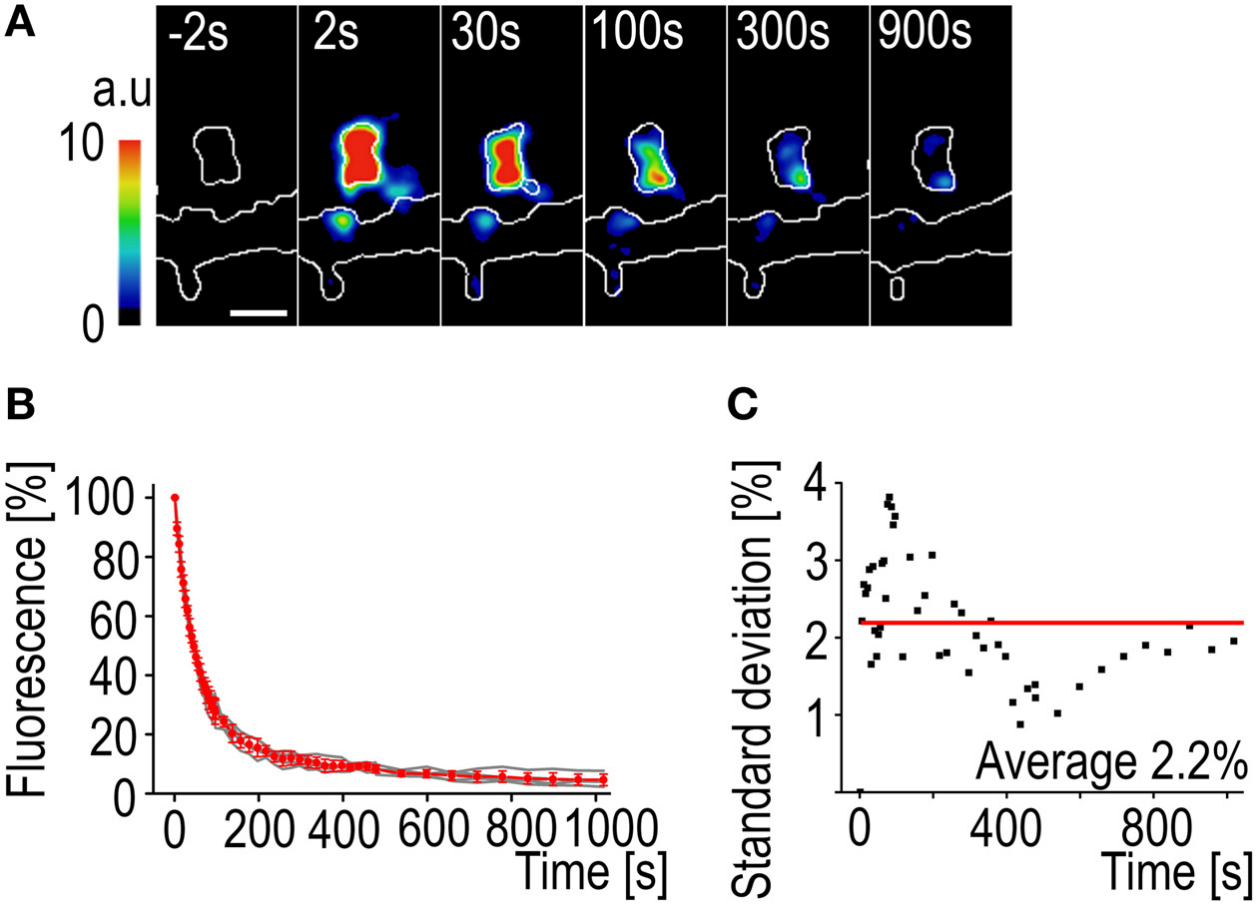
**PAGFP fluorescence decay standard deviation**. **(A)** A time-lapse series of a DIV14 rat hippocampal neuron transfected with PAGFP-actin and mCherry. The numbers represent time after photo-activation. The image is pseudocolored according to the scale on the left. The outlines of the cell were derived from mCherry fluorescence. Scale bar, 1 μm. **(B)** The PAGFP-actin fluorescence decay data from DIV14 hippocampal neuron spines. The gray traces represent individual spines and the red mean ± SD, *n* = 5 spines. **(C)** The standard deviation of the mean PAGFP fluorescence decay curve from **(B)**. The red line represents the average of 2.2%.

The most notable difference in using PAGFP decay instead of FRAP to measure the actin turnover rate is the smaller variation in fluorescence change rate. The activation of the PAGFP only requires a very short, millisecond-range, pulse of the 405 nm laser. The fast activation results in a “freeze frame” image of the F-actin at one moment and the observed decay is only affected by the filament depolymerization and disassembly rates (Figures 4A,B). This can be seen as the low average standard deviation of the decay curves (2.2%) (Figure 4C). It is also possible to follow fluorescence decay much longer than fluorescence recovery. The decaying fluorescence signal does not need to compete with intrinsic signal fluctuation so a stable recording can be maintained even at very low levels of fluorescence.

#### Analysis of the PAGFP fluorescence decay data

Due to the stable decay characteristics of the PAGFP-actin fluorescence individual measurements can be well fitted to an equation. The longer recordings also make it possible to determine the turnover rate for the slowly recovering actin pool. Additionally, no change in spine size was observed following PAGFP activation with 405 nm laser (mean spine size was 0.98 ± 0.083 μm before and 0.88 ± 0.086 μm after activation, *p* > 0.05, paired *t*-test).

**Figure 5:**
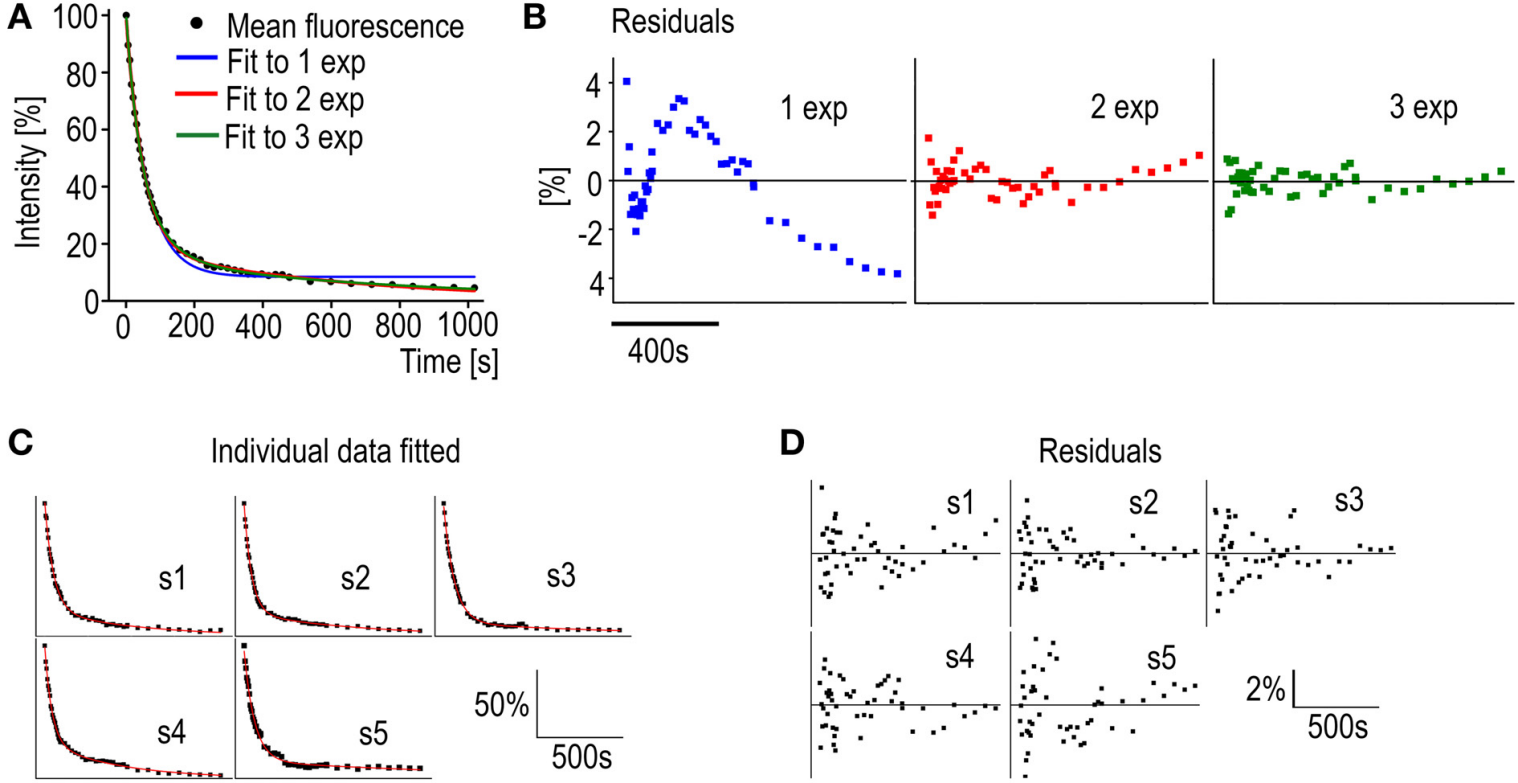
**Analysis of PAGFP decay curves**. **(A)** Averaged PAGFP-actin fluorescence data from DIV14 rat hippocampal neuron spines fitted to equations with a different number of components. Blue trace = 1 component equation, red trace = 2 component equation and green trace = 3 component equation. *n* = 5 spines. **(B)** The regular residual means from the fit curves from **(A)**. **(C)** Individual PAGFP fluorescence decay data measured from DIV14 rat hippocampal neuron spines fitted to the 2-component equation as in **(A)**. Curves were named as spines (s) 1–5. **(D)** Residuals corresponding to the fits in **(C)**.

Before fitting the individual spine data to an equation the number of differently regulated components can be evaluated. A mean decay curve from several measured spines of similar sizes (spine head width 0.98 ± 0.083 μm) was fitted to single-, bi-, or triexponential decay equations (Figure 5A). The accuracy of the fits can be estimated from the mean residuals (Figure 5B). First the distribution of the residuals was examined. All residual are normally distributed (Shapiro-Wilk test significance values >0.05) and therefore fulfill the first criterion. In Figure 5B it is seen that when the mean curve is fitted to a one-component exponential decay equation the residuals are not randomly distributed along the timescale. This indicates a poor fit. When a biexponential decay equation is used, the residuals are more randomly distributed along the timescale except for a few final values, which might indicate a third even slower turnover rate component. Using a triexponential decay equation does lead to randomly distributed residuals with smaller values but the standard errors of the time constant values increase (Table 3).

**Table 3:** **PAGFP-actin averaged data fitted to one $\left(y(t)=y_{0}+Ae^{-\frac{t}{t_{1}}}\right)$, two $\left(y\left(t\right)=A_{1}e^{-\frac{t}{t_{1}}}+\;A_{2}e^{-\frac{t}{t_{2}}}\right)$ and three $\left(y\left(t\right)=A_{1}e^{-\frac{t}{t_{1}}}+\;A_{2}e^{-\frac{t}{t_{2}}}+\;A_{3}e^{-\frac{t}{t_{3}}}\right)$ -component equations**.

**Parameter**		**1 exp**	**2 exp**	**3 exp**
y_0_^*^	Value	8.4	–	–
y_0_	*SE*	0.42	–	–
A_1_	Value	89	82	57
A_1_	*SE*	1.2	0.62	17
t_1_	Value	62	50	40
t_1_	*SE*	1.6	0.74	5.0
A_2_	Value		18	30
A_2_	*SE*		0.61	16
t_2_	Value		630	91
t_2_	*SE*		32	28
A_3_	Value			14
A_3_	*SE*			1.5
t_3_	Value			830
t_3_	*SE*			110
Statistics	Reduced Chi-Sqr	4.0262	0.3950	0.2552
Statistics	Adj. R-Square	0.9937	0.9994	0.9996

*A_x_ represents the component coefficient and t_x_ the time constant, inverse of the rate constant*.

* *y_0_ is a constant depicting an immobile pool used only in fitting to 1 exp due to the curve not reaching zero*.

Moreover, the two faster component time-constants in three component fit are fairly close to each other (40 vs. 91 s) and as such do not present new biological insight and can be seen as overfitting. These observations, taken together with the prior knowledge from Honkura et al. (2008) of the two differently regulated components of spine F-actin, led us to conclude that, with measurements of this timescale, the decay of the PAGFP-actin fluorescence from spines is best described by an equation of two differently regulated components.

Fitting the data from individual measurements resulted in a mean stable component size of 18 ± 5.8% as well as mean time constants of 51 ± 8.4 s and 840 ± 390 s for the dynamic and stable components, respectively (Figure 5C, Table 4). The residuals of the fits confirm the low noise level of the measurements (Figure 5D); the residuals spread between −4 and 4%, with a mean absolute value of 1.1 ± 0.84%. This range is five-fold smaller than in FRAP measurements. The narrower range of time constants allows for a lower number of *n* to be used. To detect a 6 s difference in dynamic pool time constants (6 s is percentually the same order change from PAGFP-obtained time constant as 2 s is in FRAP-obtained time constants) with a 90% statistical power only requires an *n* of 17 measurements.

**Table 4:** **PAGFP data curve fitted to the two-component equation $\left(y\left(t\right)=A_{1}e^{-\frac{t}{t_{1}}}+\;A_{2}e^{-\frac{t}{t_{2}}}\right)$ where A_1_ and A_2_ are the coefficients of the components and t_1_ and t_2_ the time constants**.

**Spine**	**A_1_ Value**	**A_1_ SE**	**t_1_ Value**	**t_1_ SE**	**A_2_ Value**	**A_2_ SE**	**t_2_ Value**	**t_2_ SE**	**Statistics Reduced Chi-Sqr**	**Statistics Adj. R-Square**
S1	82.3	1.41	50.0	1.60	20.0	1.43	522	50.9	1.656	0.9976
S2	83.4	1.00	43.4	1.08	17.6	0.912	671	59.6	1.265	0.9979
S3	89.0	1.14	61.3	1.56	10.3	1.06	1150	278	1.780	0.9975
S4	73.6	1.33	41.1	1.46	27.3	1.35	422	26.4	1.479	0.9976
S5	84.0	1.53	60.4	2.24	13.4	1.37	1440	425	3.656	0.9942
Average	82.5	1.28	51.2	1.59	17.7	1.22	841	168	1.967	0.9969
SD	4.97	0.190	8.38	0.374	5.82	0.202	390	157	0.8622	0.001374

## Measuring the actin filament organization in dendritic spines

**Figure 6:**
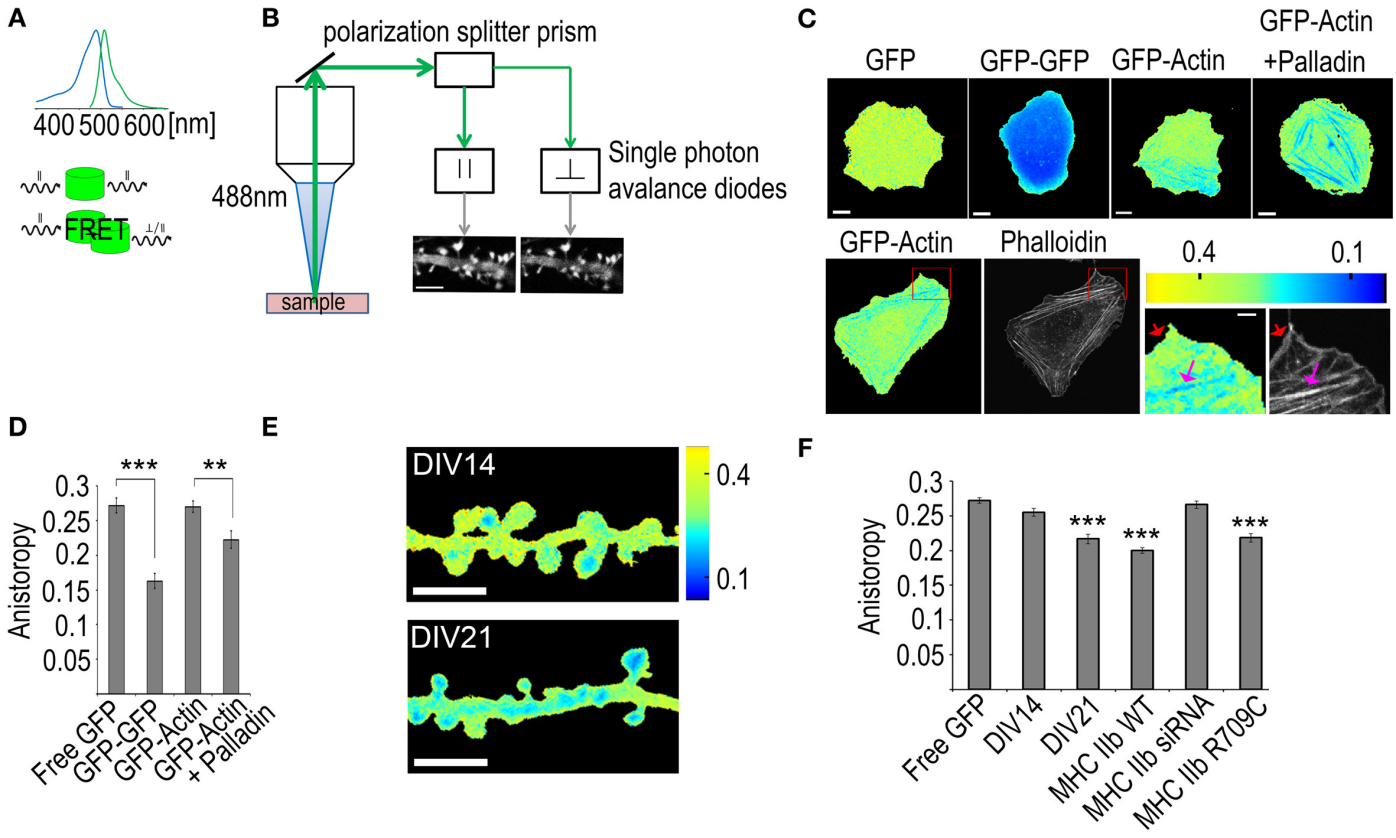
**Using fluorescence anisotropy measurements to study actin bundling**. **(A)** Upper panel: the absorption and emission spectra of GFP. Lower panel: a schematic of the differences between direct emission and FRET-mediated emission where the polarization information is lost. **(B)** The signal path of the emission. The emission signal is first directed to a polarization splitter prism that separates the signal having polarization parallel to the excitation laser from the perpendicular one. The signal is then directed to two single photon avalanche diode detectors that form accurate intensity images of the sample. Scale bar, 5 μm. **(C)** Upper panel: Fluorescence anisotropy images of U2OS cells transfected with GFP, GFP-GFP, GFP-actin or GFP-actin + palladin-mCherry, Scale bars 10 μm. Lower panel: first frame: a fluorescence anisotropy image of a U2OS cell transfected with GFP-actin, second frame: the same cell with F-actin stained with phalloidin-Alexa 594. The two last frames: enlargements of the area indicated by the red rectangle in the fluorescence anisotropy and the phalloidin images, red arrows indicate the non-bundled F-actin in the cell cortex and the pink arrows indicate the bundled stress fibers. Scale bars: whole cell images 10 μm, enlarged sections 2 μm. All the fluorescence anisotropy images are pseudocolored according to the scale in the upper right corner of the lower panel. **(D)** Mean GFP anisotropy values of whole U2OS cells transfected with free GFP (*n* = 18 cells), GFP-GFP (*n* = 15 cells), GFP-actin (*n* = 23 cells) or GFP-actin with palladin-mCherry (*n* = 13 cells). ANOVA shows that there are statistically significant differences between groups in fluorescence anisotropy *F*_(3, 65)_ = 24.335, *p* < 0.0001. Free GFP (*M* = 0.27) had statistically significant (*p* < 0.0001) higher values than GFP-GFP (*M* = 0.16), likewise GFP-actin (*M* = 0.27) had higher values than GFP-GFP. Free GFP had statistically significant (*p* < 0.01) higher values than GFP-actin + palladin (*M* = 0.22), likewise GFP-actin + Palladin had higher values than GFP-GFP and, GFP-actin had higher values than GFP-actin + Palladin. All graphs mean ± s.e.m. **(E)** Anisotropy images of DIV14 and DIV21 rat hippocampal neurons color-coded according to the scale on the right. Scale bar, 5 μm. **(F)** Comparison of the mean anisotropy values from dendritic spines of DIV14 hippocampal neurons or DIV21 hippocampal neurons (DIV21). DIV21 neurons were transfected with GFP-actin *n* = 66 spines. DIV14 neurons were transfected with various constructs: (1) free GFP (marked as free GFP) *n* = 49 spines, (2) GFP-actin (marked as DIV14) *n* = 77 spines, (3) GFP-actin and mCherry-MHC IIb *n* = 61 spines, (4) GFP-actin and MHC IIb siRNA *n* = 69 spines, (5) GFP-actin and mCherry-MHC IIb R709C *n* = 41 spines, (6) ANOVA showed that there are statistically significant differences in anisotropy between groups *F*_(8, 574)_ = 20.641, *p* <0.0001. DIV14 (*M* = 0.25) had higher values (*p* < 0.0001) than DIV21 (*M* = 0.21), MHC-WT (*M* = 0.20), MHC IIb R709C (*M* = 0.21). All graphs mean ± s.e.m; ^**^p < 0.01, ^***^p < 0.001.

Due to the small size of the dendritic spines, actin filament organization has been very difficult to determine. With the recently developed fluorescence anisotropy assay (Vishwasrao et al., 2012), we can measure how densely GFP molecules are packed in cells. GFP-actin fluorescence normally retains the polarization of the excitation light. This polarization information is lost when GFP-actin undergoes FRET with other nearby localized GFP-actin molecules (distance < 10 nm). The ratio of detected parallel and perpendicular emission light (anisotropy) can be used to derive information about F-actin structures (Figures 6A,B).

$$E=\frac{1}{1+{\left(\frac{r}{R_{0}}\right)}^{6}}$$

Equation 3: FRET efficiency *r* = distance between the fluorophores *R*_0_ = Förster distance.

Equation 3 represents the FRET efficiency as a function of the distance between the fluorophores. The Förster radius *R*_0_, the distance at which the energy transfer efficiency between the fluorophore pair is 50%, for GFP-GFP interaction is 4.58 nm (Vishwasrao et al., 2012). The probability for FRET to occur drops to less than 1% when the fluorophore pair is further than 10 nm apart. It is also indicated in the Vishwasrao et al. (2012) that in compartments of dense actin the inter-filamentary FRET can be equal or dominant form of energy transfer.

We wanted to evaluate the method and to investigate whether the signal observed is the result of intra- or inter-filament interactions of GFP-actin molecules. It is important to note that GFP-actin expression levels 24 h after transfection are typically low [6% of total actin has been estimated to be GFP-labeled (Westphal et al., 1997)], which is important to minimize over-expression artifacts and improve overall neuronal health. We estimated that under these conditions maximally every 10th actin monomer carries GFP. Thus, the probability for intra-filament FRET is low although not impossible (Vishwasrao et al., 2012). We conclude that the mathematical reasoning strongly supports the hypothesis that with fluorescence anisotropy we mainly detect inter-filament FRET.

To experimentally test whether the fluorescence anisotropy measures intra- or inter-filament FRET, we first calibrated the fluorescence anisotropy boarder values from U2 osteosarcoma cells (U2OS) expressing either free GFP or GFP dimers [two GFP molecules fused together via a molecular linker allowing for efficient energy transfer between fluorophores (Dopie et al., 2012)]. The free GFP resulted in average values of 0.27 (dimensionless ratio) whereas the GFP dimers resulted in a value of 0.16 (Figures 6C,D). Thus, with GFP-actin we should obtain values within this range, where values close to 0.27 indicate actin monomers or actin filaments where two GFP molecules do not get close enough for FRET to occur. We next tested the fluorescence anisotropy values in U2OS cells expressing GFP-actin. Low anisotropy values were only detected along stress fibers (Figure 6C). Stress fibers are tightly cross-linked thick bundles of myosin II and actin filaments. To further enhance actin cross-linking and to test if enhanced cross-linking can decrease the fluorescence anisotropy, we over-expressed palladin, an actin bundling protein, the over-expression of which leads to the formation of unusually robust actin-stress fibers in cells (Rachlin and Otey, 2006; Dixon et al., 2008). The whole cell fluorescence anisotropy was decreased in cells expressing palladin together with GFP-actin (0.27 vs. 0.22, respectively) (Figures 6C,D). Stress fibers could be clearly distinguished from the fluorescence anisotropy images of cells expressing GFP-actin with palladin (Figure 6C). We further tested the idea of measuring actin bundling with fluorescence anisotropy by comparing the fluorescence anisotropy values in lamellipodia and along stress fibers (Figure 6C). Lamellipodia are composed of actin filament networks. These networks are densely packed but filaments are not aligned as bundles (Svitkina and Borisy, 1999). We first obtained images of GFP-actin anisotropy from live U2OS cells that were subsequently fixed and the F-actin was visualized with phalloidin staining (Figure 6C, lower panel). The phalloidin staining shows clearly both lamellipodium (red arrow) as well as stress fibers (pink arrow). Anisotropy decreases along stress fibers but not in the lamellipodium (Figure 6C, lower panel). The anisotropy results indicate that the anisotropy decreases significantly only when actin filaments are tightly bundled together, as in stress fibers. Taken together, these results indicate that in cells expressing low levels of GFP-actin the anisotropy values represent the level of actin filament bundling. The fact that the average fluorescence anisotropy value in the area of lamellipodium with a lot of F-actin was similar to values detected with free GFP indicates that the intra-filament FRET is actually under the detection level. Thus, we conclude that fluorescence anisotropy is a potentially highly useful tool for actin bundling measurements.

We next applied this method to analyze how actin filament organization changes during neuronal maturation. We first measured the mean anisotropy from the dendritic spines of neurons transfected with GFP-actin at DIV14 and DIV21 and found the mean anisotropy from DIV21 neuron spines to be significantly lower than that from DIV14 neuron spines (0.22 vs. 0.26, respectively) (Figures 6E,F), indicating an increase in actin bundling. This is consistent with our previous study, where we described—using FRAP and PAGFP fluorescence decay—that the size of the F-actin stable pool increases during neuronal maturation and hypothesized that the stable F-actin pool is increased by enhanced cross-linking of the actin filaments (Koskinen et al., 2014). Furthermore, no significant difference was found between DIV14 anisotropy values and free GFP (0.26 and 0.27 respectively) (Figure 6F), indicating very low levels of bundled actin in immature spines. This also indicates that F-actin polymerization itself is not sufficient to decrease the anisotropy values. Based on our previous FRAP and PAGFP fluorescence decay results, we proposed that myosin IIb cross-links the actin filaments in dendritic spines independent of its motor activity. Thus, to further test the fluorescence anisotropy method, we tested how myosin IIb over-expression, depletion and expression of the non-contractile myosin IIb mutant construct (R709C) affect fluorescence anisotropy values. The expression of MHC IIb or MHC IIb R709C both resulted in significantly lower levels of anisotropy (0.20 and 0.22, respectively) compared to control, suggesting their function in actin cross-linking. Furthermore, no significant difference was found between control neurons and MHC siRNA-transfected ones (0.26 and 0.27 respectively) (Figure 6F), indicating no or very few cross-linked actin filaments in myosin IIb depleted DIV14 neurons. This data fits well with our previous FRAP and PAGFP fluorescence decay data showing that myosin IIb overexpression (both wild-type and non-contractile mutant constract) significantly increased the size of the stable actin pool while the depletion of myosin IIb using siRNA completely abolished the stable actin pool (Koskinen et al., 2014).

With fluorescence anisotropy we can measure how closely GFP molecules are packed in cells in different cell areas. Our mathematical reasoning as well as experimental evidence suggest that this method measures mainly the GFP-actin:GFP-actin FRET between closely packed actin filaments, and thus can be used as a method to measure values for actin filament bundling. The closer the actin filaments are to each other, the smaller is the fluorescence anisotropy value.

## Discussion

FRAP has been extensively used and refined in studies of actin dynamics especially in fibroblasts. So far only a few studies utilized this method for studying actin dynamics in neurons and especially dendritic spines, but it is clear that there is an increasing interest. It is, therefore, worthwhile to consider what parameters need to be taken into account to get the best results. The main drawbacks of using FRAP in dendritic spines are the large variation between measurements, fast dendritic spine dynamics and changes in GFP-actin intensity as well as spine enlargement following photo-bleaching (Figures 1B,D). Currently, the reasons underlying spine enlargement are not clear but it is plausible that photo-bleaching increases the rate of actin polymerization. High laser power illumination can cause severing of actin filaments and rapid creation of new barbed ends (Jacobson et al., 2008; Reymann et al., 2011). This could explain the rapid increase in spine size, the overshoot of the fluorescence from pre-bleach levels and the high fluctuation of the post-bleaching fluorescence. If this is the case and the newly formed barbed ends lead to increased net polymerization of the filaments then the rate constants obtained using FRAP overestimate the turnover rate of the dynamic actin pool. It seems that this effect is not uniformly manifested across all conditions or imaging systems. Manipulating ABP levels or activity seems to affect the magnitude of the observed effect. For example, the knockdown of a capping protein could lead to a prolonged increase in bulk polymerization compared to control due to the inability to control the polymerization by capping the newly formed filament barbed ends. This emphasizes the need to control and test the laser power used for photo-bleaching. There have been attempts to improve the method, such as dual color FRAP (Dunn et al., 2002). Use of two colors could be useful also in spines. The reference color would allow normalization against intrinsic fluctuations of the dendritic spine of interest.

In control conditions and especially in young neurons, the stable actin pool often comprises only a few percent of the total F-actin, depending on spine size (Star et al., 2002; Honkura et al., 2008). Due to GFP-actin fluorescence fluctuation, particularly when the recovery has reached the plateau level (Figure 1B), the size and the turnover rate of the stable actin pool are difficult to determine from single FRAP measurements. Averaging frees the analysis from many of the problems posed by high fluctuation of the values (Figures 3B–F). On the other hand, averaging makes statistical testing impossible. It could be useful to look into methods to estimate how “different” two average curves are. One such method is the Kullback-Leiber divergence (Kullback and Leibler, 1951). However, using this method to compare two curves does not directly lead to a *p*-value but the significance is estimated by other means. More elaborate means of analysis are also found where fitting is performed to a stochastic model of actin treadmilling (Halavatyi et al., 2010). These models work well in *in silico* simulations but fitting measured data to six different parameters without fixed values is not feasible.

Using PAGFP-actin fluorescence decay has the advantage of a more stable signal compared to FRAP experiments in dendritic spines (Figure 3). The brief, moderate intensity, pulse needed for the photo-activation minimizes the adverse effects of laser illumination. The stable recordings are easy to fit to an equation. This allows to treat spines as individual units and compare several parameters, such as spine head size, in addition to the actin turnover data. Compared to FRAP, the PAGFP fluorescence decay is a bit more difficult technique to use. It requires the co-transfection of another fluorophore to visualize the cell and more optimization is needed to determine the expression level of the PAGFP-actin and to standardize imaging settings.

One of the main questions regarding the actin turnover is the distinction between filament treadmilling and actin structure assembly/disassembly. This question has been addressed in studies of actin dynamics in lamellipodia, a structure consisting of highly dynamic actin filament network (McGrath et al., 1998; Lai et al., 2008; Miyoshi and Watanabe, 2013; Smith et al., 2013). According to models based on single molecule tracking results it is unlikely that filament treadmilling alone is responsible for the fast turnover of actin structures in lamellipodia (Miyoshi and Watanabe, 2013). A new type of actin structure, a slow diffusing oligomere, resulting from a cleavage of an actin filament, which polymerizes to filaments along the lamellipodia and itself depolymerizes according to a distinct rate has been proposed as an explanation for the discrepancy between single molecule tracking and bulk kinetics experiments (Smith et al., 2013). It is plausible that rebinding of actin monomers/oligomers also happens to some extend in dendritic spines. This should be taken into considerations if absolute values of the structure turnover rate are needed. It is also possible that the rebinding of monomers and oligomers are at least partly responsible for the stable component observed in FRAP and PAGFP decay experiments.

Okamoto et al. (2004) first took advantage of FRET between actin monomers labeled with cyan fluorescent protein (CFP) and yellow fluorescent protein (YFP) in living neurons. The method used to measure energy transfer was fluorescence intensity based. Increase in energy transfer was interpreted as a shift in the G-actin/F-actin ratio. This was based on the assumption that energy transfer was only possible if CFP- and YFP-actin were found one after another in filamentous actin. For this to happen, at a noticeable frequency, the amount of labeled actin inside the cell must be very high (Vishwasrao et al., 2012). Also, FRET between two GFP fluorophores has been proposed as a method to measure the level of actin polymerization (Vishwasrao et al., 2012). Here, we propose a novel idea that these FRET assays would be more suitable to study the level of actin filament bundling instead of actin polymerization. Although the method is theoretically feasible and our experiments support this idea, it is important to note that in cells the intra- and inter-filament FRET cannot be completely separated as they form a continuum along the labeling density. Nevertheless, the probability of intra-filament FRET is completely determined by a sufficient labeling density of the individual filaments. Inter-filament FRET, on the other hand, depends on the bundlling of filaments. Increase of bundling will always result in an increase in local F-actin concentration and therefore units of GFP-actin inside a given volume, without an increase in labeling density of individual filaments. Furthermore, it is difficult to produce direct evidence for actin bundling vs. polymerization in cells because we can neither block polymerization without affecting actin bundles (no filaments—no bundles) nor can we block bundling without affecting the G-/F-actin ratio (if actin filaments are not bundled they are more vulnerable for cofilin induced depolymerization). Therefore, the methodological development would benefit from *in vitro* evaluation, such as actin *in vitro* polymerization and bundling assays. Regardless of whether fluorescence anisotropy measures polymerization or bundling, our results show that actin monomers are more closely packed in dendritic spines of 3 week old neurons, compared to 2-week old neurons. Based on our earlier results (Koskinen et al., 2014) and other literature, it is plausible that this tighter packing of actin reflects a higher level of actin filament bundling.

Although GFP-actin has widely been used for live-cell imaging in various model systems (Fischer et al., 1998; Schneider et al., 2002; Honkura et al., 2008), in all methods utilizing GFP-actin it is important to remember that especially highly over-expressed GFP-actin may influence actin dynamics (Aizawa et al., 1997; Feng et al., 2005; Deibler et al., 2011). Moreover, GFP-actin might be excluded from formin-assembled actin filaments, as GFP-actin expressed in fission yeast does not incorporate into the contractile ring assembled primarily by formin homology protein Cdc12p (Chen et al., 2012). Formins play critical roles in assembling various actin structures, including tightly bundled stress fibers in fibroblasts (Hotulainen and Lappalainen, 2006; Tojkander et al., 2011). Thus, it is possible that the fluorescence anisotropy will overlook some formin assembled actin bundles. Similarly, FRAP and photoactivation assays may ignore formin-based actin dynamics.

## Conclusions

FRAP is a fast and easy technique to use but - especially in dendritic spines—there are aspects that need to be considered in its application and analysis. The bleaching laser pulse may have its own effect of increasing spine size and dynamics. As such, FRAP is most suitable for studying differences in dynamic pool turnover rate. In addition, FRAP can detect clear differences in the sizes of the F-actin stable pool. The use of a dual color approach could help to tackle the difficulties but is technically more challenging. Single FRAP measurements are difficult to fit to equations and several measurements need to be taken in order to obtain reliable results. The comparison of two average curves from FRAP measurements seems to be the best way to show the differences between test groups or conditions but demonstrating the statistical significance requires advanced statistics.

PAGFP fluorescence decay offers the possibility for longer, more stable measurements. PAGFP fluorescence decay is especially useful for measuring the size and turnover rate of the stable actin pool. Small differences in dynamic pool turn-over rate may be lost due to longer time interval in imaging often utilized compared to FRAP. The big advantage of this method is that the measurements can be analyzed individually and there is much less variance in decay measurements than in FRAP measurements. The possibility of individual spine measurements without averaging enables comparison of single spines to one another.

Fluorescence anisotropy is an easy and fast method. Fluorescence anisotropy is particularly useful in providing information about structures that can not be examined in detail using conventional methods, such as dendritic spines.

If only one method is used to measure actin turnover rate, PAGFP fluorescence decay seems to be more reliable than FRAP. However, it is important to remember that FRAP is measuring the F-actin assembly whereas PAGFP decay is measuring the F-actin disassembly. Thus, these methods complement each other and should optimally be used together. In combination with FRAP and PAGFP decay, fluorescence anisotropy provides an additional method to improve our understanding on the organization and dynamics of dendritic spine actin filaments.

## Author contributions

Mikko Koskinen and Pirta Hotulainen designed the experiments and wrote the manuscript. Mikko Koskinen performed the experiments.

### Conflict of interest statement

The authors declare that the research was conducted in the absence of any commercial or financial relationships that could be construed as a potential conflict of interest.
